# Mechanisms Underlying Pneumococcal Transmission and Factors Influencing Host-Pneumococcus Interaction: A Review

**DOI:** 10.3389/fcimb.2021.639450

**Published:** 2021-04-28

**Authors:** Ayumi Morimura, Shigeto Hamaguchi, Yukihiro Akeda, Kazunori Tomono

**Affiliations:** ^1^ Department of Infection Control and Prevention, Osaka University Graduate School of Medicine, Osaka, Japan; ^2^ Division of Infection Control and Prevention, Osaka University Hospital, Osaka, Japan; ^3^ Research Institute for Microbial Diseases, Osaka University, Osaka, Japan

**Keywords:** *Streptococcus pneumoniae*, animal models, bacterial transmission, pneumococcal transmission, bacterial shedding

## Abstract

*Streptococcus pneumoniae* (also called pneumococcus) is not only a commensal that frequently colonizes the human upper respiratory tract but also a pathogen that causes pneumonia, sepsis, and meningitis. The mechanism of pneumococcal infection has been extensively studied, but the process of transmission has not been fully elucidated because of the lack of tractable animal models. Novel animal models of transmission have enabled further progress in investigating pneumococcal transmission mechanisms including the processes such as pneumococcal shedding, survival in the external environment, and adherence to the nasopharynx of a new host. Herein, we present a review on these animal models, recent research findings about pneumococcal transmission, and factors influencing the host-pneumococcus interaction.

## Introduction

*Streptococcus pneumoniae* (also known as pneumococcus) is a commensal that colonizes the upper respiratory tract and a pathogen that causes invasive diseases such as otitis media, pneumonia, sepsis, and meningitis. Pneumococcus is a gram-positive bacterium first isolated in 1881 (Bennet et al., 2015) with 100 distinct serotypes (Ganaie et al., 2020). Despite advances in therapeutics and vaccines, the world continues to experience a high burden of the disease, especially in the vulnerable populations which include young children, older adults, and immunocompromised persons (Centers for Disease Control and Prevention, 2017). In 2017, the World Health Organization published a list of antibiotic-resistant “priority pathogens” which included penicillin-nonsusceptible pneumococcus as one of the 12 families of bacteria that pose the greatest threat to human health (WHO, 2017). Multidrug resistance, defined as resistance to more than any three antimicrobial agents of different classes, was observed in 59.3% of isolates from Asian countries (Kim et al., 2012). As the problem of pneumococcal resistance to antibiotics worsens, the effectiveness of vaccines becomes even more important (Kim et al., 2016). Currently available pneumococcal vaccines include the 23-valent pneumococcal polysaccharide vaccine (PPSV23) and the 10-valent or 13-valent pneumococcal protein-conjugate vaccine (PCV10, PCV13) which replaced the PCV7. These three vaccines, especially the PCV, have significantly reduced the incidence of invasive pneumococcal disease, and pneumococcal pneumonia (Fine et al., 1994; Simonsen et al., 2011; Suzuki et al., 2017).

To effectively prevent pneumococcal disease, it is important to understand the natural course of infection. Pneumococcal infection can be divided into three stages: transmission, colonization, and invasion (Weiser et al., 2018). To date, various studies have been conducted to understand various aspects of the mechanism of pneumococcal colonization and invasion; however, the process of transmission was not elucidated until the establishment of novel animal models. We present a review on the animal models that have been used to study disease transmission, the underlying mechanism, and factors that influence the host-pneumococcus interaction.

## Establishment of Tractable Animal Models to Study Pneumococcal Transmission

**Figure 1:**
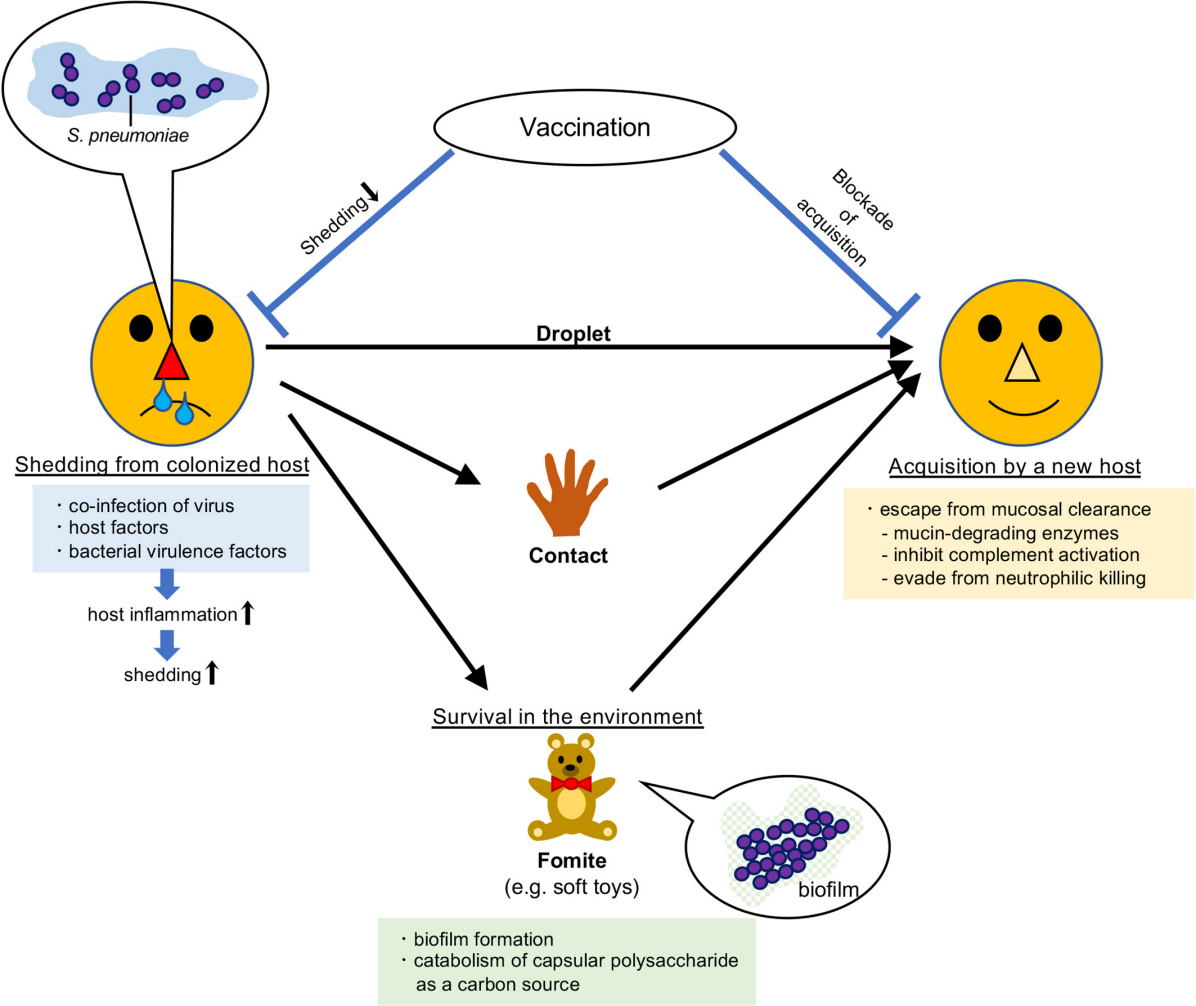
Route of transmission of *Streptococcus pneumoniae*. *Streptococcus pneumoniae* colonizes the mucosal surface of the upper respiratory tract. It can be transmitted to new hosts by droplets, direct contact, or *via* fomites. Vaccination prevents its transmission.

For pneumococcal transmission to new hosts, it first has to leave the colonized host (shedding). It should then survive in the environment before reaching the new host, unless it is transmitted through droplets or direct contact. Finally, it needs to be acquired successfully by the new host without being eliminated (**Figure 1**). To examine the mechanisms of each process, establishing tractable animal models is indispensable.

Experimental investigation on pneumococcal transmission has a relatively short history because animal models have only recently become available. There are only a few tractable animal models other than *Streptococcus pneumoniae* for bacterial transmission. These include an infant rat model of intralitter *Haemophilus influenzae* transmission (Halsey et al., 1980) and a possible transmission model of *Salmonella* species: a murine model of chronic *Salmonella* carriage in the gallbladder using *Salmonella enterica* subsp. *enterica* serovar Typhimurium (Gonzalez-Escobedo and Gunn, 2013). In addition, a murine model of *Klebsiella pneumoniae* transmission through the fecal-oral route has recently been described (Young et al., 2020). Epidemiological studies suggest that pneumococcal transmission requires close contact and is more likely to occur in the setting of viral coinfection (Gwaltney et al., 1975). Among respiratory viruses, influenza A virus (IAV) is known to be associated with severe secondary pneumococcal pneumonia (Morens et al., 2008). In contrast, severe secondary pneumococcal pneumonia is less common among people severe acute respiratory syndrome coronavirus 2 (SARS-CoV-2) infection (Fattorini et al., 2020; Lansbury et al., 2020; Zhu et al., 2020; Garcia-Vidal et al., 2021), although its severity and poor prognosis are suggested; a severe case has been reported in an infant (Nieto-Moro et al., 2020) and high mortality rate in the elderly patients is also reported (Rodriguez-Nava et al., 2020). Surveys of children attending daycare centers have shown that respiratory viral infection and symptoms of rhinitis are associated with bacterial colonization and transmission (Rodrigues et al., 2013; Thors et al., 2019).

As for the pneumococcal transmission animal model, in 1998, it was reported that pretreatment with pneumococcal polysaccharide immunoglobulin led to a significant reduction in nasal colonization in an intralitter transmission model of infant rats (Malley et al., 1998). However, there are no subsequent reports of this model being used.

In 2010, two types of animal transmission models were reported. McCullers et al. demonstrated that aerosol transmission of pneumococcus occurred among adult ferrets that were coinfected with IAV (McCullers et al., 2010). In this model, the ferrets were infected with IAV or *S. pneumoniae* intranasally. They examined four combinations of pairs of ferret infection (the donor ferrets with pneumococcal infection with or without IAV coinfection, cohoused with the contact ferrets infected with IAV or not) at three different distances (the same cage, 3-feet separation, and 10-feet separation). As a result, donors with prior IAV infection were more susceptible to pneumococcal transmission and disease and that of contact ferrets increased their susceptibility to pneumococcal acquisition. In another report, it was shown that increased pneumococcal colonization and disease in the presence of IAV using the model of infant mice colonized with *S. pneumoniae* and subsequently infected with IAV 3 days later (Diavatopoulos et al., 2010). In this model, a litter of 5-day-old C57BL/6 mice was randomly divided into 2 equally sized groups, “index” mice and “contact” mice, and index mice were intranasally colonized with *S. pneumoniae* and infected with IAV 3 days later. Secondary infection with IAV resulted in a strong increase in pneumococcal load and its transmission to contact pups. This model has been of great use for further analyses of both bacterial factors under the condition of transmission. However, the presence of IAV is essential for pneumococcal transmission in this model, which raises the problem that host reactions against pneumococci and IAV are difficult to discern.

In 2016, Zafar et al. developed a murine transmission model without a respiratory virus coinfection, which can be utilized to examine bacterial and host factors that contribute to pneumococcal transmission free from the effects of viral coinfection (Zafar et al., 2016). Four-day-old pups were inoculated intranasally with *S. pneumoniae* without anesthesia, and their pneumococcal shedding from the upper respiratory tract was quantified. This model of pneumococcal transmission was adapted to an infant mouse model of Group A *Streptococcu*s (Vega et al., 2020).

In summary, these tractable pneumococcal transmission animal models with or without IAV coinfection have enabled researchers to examine the transmission process in detail since the last decade (**Table 1**). As shown, the models with IAV coinfection are the first animal models of pneumococcal transmission, but the strong inflammation caused by IAV infection makes it difficult to evaluate the host response to pneumococcal infection alone. The pneumococcal monoinfection model is a breakthrough which established pneumococcal transmission independent from IAV, and showed the method of measuring the quantity of shedding.

**Table 1:** Remarkable animal transmission models of *Streptococcus pneumoniae*.

Animal type	Co-infection	Transmission	Characteristics	Author	Year
ferret	IAV	co-housed pairs of ferrets	-similar airway symptoms to human -difficult to exclude the influence of IAV -not genetically homogenous	McCullers et al.	2010
infant mouse	IAV	intralitter transmission	-easier to handle than ferrets -difficult to exclude the influence of IAV -genetically homogenous	Diavatopoulos et al.	2010
infant mouse	none	intralitter transmission	-easier to handle than ferrets -can evaluate the effect of pneumococcal infection solely -quantitative evaluation of pneumococcal shedding -no adult models	Zafar et al.	2016

## Shedding

The IAV coinfected infant mouse model enabled researchers to assess the effect of inflammation caused by IAV infection on *S. pneumoniae* transmission. IAV infection renders the mucosal surface of the human respiratory tract suitable for pneumococcal proliferation by decreasing mucociliary clearance (Levandowski et al., 1985), enhancing the expression of glycoproteins within mucus (Barbier et al., 2012) and providing sialic acid as a nutrient source (Siegel et al., 2014). Inflammation induced by IAV infection had been demonstrated to promote pneumococcal shedding from index mice at or above a level sufficient to infect uninoculated contact mice by adapting the infant mouse model coinfected with IAV (Richard et al., 2014). In this study, toll-like receptor (TLR) 2 deficiency induced more frequent transmission because of a weakened antiviral response that made the mice more susceptible to IAV infection and caused heightened inflammation. Therefore, increased inflammation is associated with increased transmission rate of *S. pneumoniae* by infected hosts. Furthermore, it was reported that a sufficiently large number of pneumococci on the mucosal surface seemed necessary for a single organism to reach the nasal mucosa of a new host because transmission rates correlate with the density of shed organisms using the infant mouse coinfected with IAV model (Kono et al., 2016).

In the infant mouse *S. pneumoniae* monoinfection model of transmission established by Zafar et al., the level of pneumococcal shedding was highest in the pups infected intranasally at age 4 days and this level depended on colonization density and pneumococcal capsule type. To achieve a high (29%) transmission rate, transmission experiments were performed with a 1:1 ratio of index mice to contact mice (Zafar et al., 2016). Both the type and the amount of capsular polysaccharide (CPS) were demonstrated to be determinants of the spread of pneumococci from one host to another with isogenic capsule switch and *cps* promoter switch mutants (Zafar et al., 2017a). In this study, the importance of CPS in shedding was revealed based on the finding that the shedding of a type 23F isolate was significantly less than that of a type 4 isolate, TIGR4 (T4), even though the colonization levels in the nasopharynx were equivalent. The T4 isolate expressing a 23F capsule showed less shedding than that of a 23F isolate expressing a type 4 capsule, and the proportion of high shedding events of the 23F isolate expressed the type 4 CPS increased compared to that of the 23F isolate. In addition, comparing strains of different genetic backgrounds expressing the same capsule type showed that genetic background had little influence on shedding. These findings suggested that CPS type is a more important factor for shedding than genetic background. They also created constructs of the same type that expressed different levels of CPS by switching the *cps* promoter region. They demonstrated that the strain with a weaker *cps* promoter showed significantly reduced shedding and transmission in the model of transmission by infant mice coinfected with IAV. A mucin-binding assay showed that the strains with the CPS type or amount whose shedding was reduced had increased mucin-binding affinity and were more strongly immobilized, suggesting a correlation between the ability to escape from mucus entrapment and pneumococcal shedding. Furthermore, using the infant mouse pneumococcal monoinfection model, two notable effects of a pneumococcal pro-inflammatory pore-forming toxin, pneumolysin (Ply) were demonstrated (Zafar et al., 2017b). First, Ply promotes inflammation, which increases shedding and enables intralitter transmission. Second, Ply increases bacterial survival outside the host. probably because Ply-induced host cell lysis helps pneumococcal survival and growth nutritionally. These findings suggest that sufficient levels of Ply are needed for pneumococcal shedding for transmission to occur. In 2019, it was demonstrated that the *dlt* locus, which is involved in D-alanilation of lipoteichoic acids (LTA) increasing TLR2-mediated inflammation and resistance to antimicrobial peptides, also plays a key role in pneumococcal shedding, using the infant mouse model (Zafar et al., 2019).

Rowe et al. described other pneumococcal genes required for effective transmission using the model of the ferret coinfected with IAV, by screening with a TnSeq library of a pneumococcal strain. They also demonstrated that targeted deletion of the putative C3-degrading protease CppA, iron transporter PiaA, or competence regulatory histidine kinase ComD significantly decreased transmissibility in the infant mouse pneumococcal monoinfection model, confirming the result of the ferret screening (Rowe et al., 2019). In particular, ComD is known to be a receptor for competence stimulating peptide (CSP) and a member of the ComABCDE pathway, which regulates competence and is associated with quorum sensing and biofilm formation (Chandler and Morrison, 1987; Håvarstein et al., 1995; Håvarstein et al., 1996; Pestova et al., 1996; Alloing et al., 1998; Ween et al., 1999; Oggioni et al., 2006). They also showed that maternal vaccination with recombinant surface-exposed PiaA and/or CppA blocked intralitter transmission and was more effective than PCV13 in this study.

Thus, it is underscored that host inflammation plays a key role in pneumococcal shedding and that bacterial factors such as capsule type and capsular amount are important, as shown by quantitative measurement of shedding in the studies using the pneumococcal monoinfection transmission model. The comprehensive genetic screening method, TnSeq, has enabled identification and quantification of pneumococcal factors required for transmission, and can be utilized to search for and prioritize vaccine candidates.

## Survival in the External Environment

Transmission can occur through exposure to fomites in addition to direct contact with carriers. For example, the teats of the dam were contaminated with large numbers of pneumococci by infected suckling mice and appeared to be the source of contagion of the pups in the mouse model, even without nasal pneumococcal colonization of the dam (Diavatopoulos et al., 2010). The duration of pneumococcal survival in the external environment ranges widely from a few hours to several months, depending on the situation, and it is longer than that of another important respiratory pathogen, *Haemophilus influenzae* (Walther and Ewald, 2004). In daycare settings, pneumococci can survive for hours and be cultured from environmental surfaces such as soft toys and crib linen (Marks et al., 2014). Furthermore, it has been reported that pneumococci have desiccation tolerance, and viable bacteria are culturable even after four weeks of desiccation at ambient temperature and humidity (Walsh and Camilli, 2011). Various clinically relevant *S. pneumoniae* strains show both survival and growth in saliva under ambient conditions (Verhagen et al., 2014). In contrast, in airway surface fluid where nutrients are limited (Barker et al., 1989; Philips et al., 2003), Ply expression of pneumococcus increases ex vivo survival, and the possible mechanism is that Ply-induced inflammation increases nutrient levels in airway secretions (Zafar et al., 2017b). Under nutrient-poor environmental conditions, pneumococci remain infectious for at least 24 hours and encapsulation prolongs bacterial survival in a serotype-dependent manner, which suggests that pneumococci catabolize their own capsular polysaccharide as a carbon source when other carbon sources are scarce (Hamaguchi et al., 2018).

Pneumococcus forms biofilms during nasopharyngeal colonization (Gilley and Orihuela, 2014). Biofilms are complex bacterial populations with an extracellular matrix (ECM) adherent to each other and/or to surfaces or interfaces (Costerton et al., 1995; Donlan and Costerton, 2002). The ECM is composed of host factors, polysaccharides, and extracellular DNA and protects bacteria from the host immune system (Moscoso et al., 2006). Pneumococci in biofilms have been shown to be less virulent than their planktonic form (Lizcano et al., 2010; Sanchez et al., 2011; Gilley and Orihuela, 2014). Marks et al. developed a novel model of biofilm on live epithelial cells both *in vitro* and *in vivo* (Marks et al., 2013). In their study, they demonstrated that infection with IAV or the host signals caused by IAV infection (febrile-range temperatures, norepinephrine, extracytoplasmic ATP, and increased nutrient availability) induced the release of bacteria from biofilms and that these dispersed bacteria appeared to be more virulent than both biofilm and broth-grown planktonic bacteria. The bacteria dispersed from biofilms showed enhanced expression of genes associated with virulence, such as the *cps* cassette, pneumolysin, adhesin PavA, and *licD2* locus involved in phosphorylcholine metabolism and promoting the opaque phenotype (Zhang et al., 1999), suggesting that high capsule expression induced high levels of inflammatory cytokines and caused more severe disease in a mouse infection model. These observations are rather phenomenological, and the precise mechanism underlying pneumococcal colony opacity phase variation, dispersal from biofilms, and upregulation of its virulence, remains unclear. Furthermore, the roles and interactions of pneumococcal biofilms, bacteria in biofilms, and hypervirulent dispersed bacteria from biofilms in viral inflammation require further investigation. Recently, it was shown that pneumococcal phase variation of colony opacity occurred by recombination of the DNA methylase genes (*hsdS*, *hsdS’*, and *hsdS’’*) in the SpnD39III and Spn556II type I restriction-modification (R-M) systems (Manso et al., 2014; Li et al., 2016). Based on these reports, Oliver et al. created phase-locked mutant strains of TIGR4 background, whose colony phenotypes were stable over multiple serial passages *in vitro* and *in vivo* (Oliver et al., 2017). In this study, it was observed that capsule expression of *hsdS* variants was less than that of the TIGR4 strain, and that the capsule expression of transparent variants was less than that of the opaque strains. In addition, the biofilm formation and viability in the biofilm of the opaque variants were reduced. Furthermore, transcriptome sequencing (RNA-seq) analyses showed that the expression levels of potential virulence factors were altered in a phase-specific manner. The concerning point of this study is its finding that transparent variants were more virulent than opaque ones, which was inconsistent with previous studies (Kim and Weiser, 1998; Kim et al., 1999). The possible reasons of this inconsistency are the difference of the capsular types (TIGR4 vs type 2, 6A, and 18C) or the route of administration (intranasal vs intraperitoneal). However, the epigenetic approach and next generation analysis such as transcriptome sequencing will enable further breakthroughs to be made in researching host-pathogen interactions.

Biofilm formation enhances the survival of pneumococci on fomites (Marks et al., 2014). The relative importance of a contagion through environmental fomites compared to that of direct contact is unclear. However, reports suggest that pneumococci in biofilms survive long enough in the external environment to transmit to new hosts. It has been shown that pneumococci remain fully infectious after an environmental exposure without nutrients for up to 24 h under experimental conditions (Hamaguchi et al., 2018). Therefore, hygiene management, such as washing hands, cleaning the surrounding areas, and disinfecting equipment, is indispensable.

## Adherence to the Nasopharynx of a New Host

To be acquired by a new host, pneumococci must escape from the host defense mechanism and adhere to the mucous membrane of the nasopharynx. Here, we focus on the pneumococcal mechanism of evading initial mucosal clearance and its impact on transmission.

First, most pneumococcal capsules are negatively charged, which is thought to enable them to evade clearance by mucus (Nelson et al., 2007). Second, pneumococci have several enzymes that degrade mucus, inhibit mucociliary clearance, and enable adhesion to the mucosa. Neuraminidase A (NanA), β-galactosidase (BgaA), and β-N-acetylglucosaminidase (StrH) sequentially deglycosylate N-linked glycans on host defense molecules (King et al., 2006). In addition, NanA and BgaA function as adhesins independently of their enzymatic activities (Uchiyama et al., 2009; Limoli et al., 2011). Furthermore, Ply released on autolysis by autolysin slows ciliary beating and inhibits mucociliary clearance (Steinfort et al., 1989; Feldman et al., 1990; Feldman et al., 2002). Recently, Fliegauf et al. discovered that pneumococci inhibit mechanical cilia-mediated clearance independently of the effect of Ply using high-speed video microscopy and live-cell imaging in a murine *in vitro* airway infection model (Fliegauf et al., 2013). Moreover, two pneumococcal proteins, namely, peptidoglycan-N-acetylglucosamine deacetylase (PgdA) and attenuator of drug resistance (Adr), modify the peptidoglycan of its cell wall and are associated with resistance to lysozyme, which cleaves bacterial peptidoglycan and has antimicrobial effects on the mucosal surface fluid (Davis et al., 2008). Pneumococcal surface protein A (PspA), is another important protein that plays an important role in invasive infections by inhibiting complement activation (Tu et al., 1999) and protecting pneumococcus from killing by apolactoferrin (Shaper et al., 2004). It has been reported that immunization with PspA is protective against pneumococcal infection in mice (Ezoe et al., 2011; Piao et al., 2014). For evasion of neutrophilic killing, pneumococcal choline-binding-protein E (CbpE, also known as Pce) decreases neutrophil recruitment by the inactivation of platelet-activating factor (PAF), a host-derived inflammatory phospholipid that stimulates neutrophil phagocytic capacity and bactericidal function (Hergott et al., 2015).

The pneumococcal zinc metalloprotease, ZmpA, cleaves immunoglobulin A1 (IgA1), the most abundant immunoglobulin on mucosal surfaces of the human upper respiratory tract, and thereby eliminates the agglutinating activity of IgA1, which prevents mechanical bacterial clearance by the mucociliary flow (Janoff et al., 2014; Roche et al., 2015). Epidemiological studies on PCVs have found that they not only protect children from invasive disease, but also prevent pneumococcal transmission to vulnerable groups, especially older adults, by reducing pneumococcal carriage among vaccinated children (herd immunity) (Whitney et al., 2003; Centers for Disease Control and Prevention, 2005; Miller et al., 2011; Whitney et al., 2014). The infant mouse model has been used to examine the role of anti-pneumococcal immunity in nasal shedding and pneumococcal transmission. It has been shown that shedding is decreased and transmission is blocked by anti-pneumococcal immunity and PCV (Zangari et al., 2017). The agglutinating function of PCV-induced IgG to type-specific pneumococcal polysaccharide appears to reduce pneumococcal shedding. In humans, the Pneumococcal Conjugate Vaccine Experimental Human Pneumococcal Carriage (PCV/EHPC) study was conducted in 2012 (Collins et al., 2015), and the EHPC model was utilized in subsequent studies. The studies revealed that pneumococcal carriage protects healthy adults against re-challenge to the homologous strain (Ferreira et al., 2013), but not against a heterologous strain type (Pennington et al., 2016). Furthermore, high levels of CPS-specific memory B cells are associated with protection against acquisition (Pennington et al., 2016) and pneumococcal agglutination of airway secretions mediated by CPS-specific antibodies after PCV vaccination appears to be a key mechanism of protection against acquisition of carriage (Mitsi et al., 2017).

*S. pneumoniae* has various strategies to escape from mucosal clearance and successfully colonize new hosts. In humans, PCV prevents not only invasive pneumococcal disease, but also the acquisition of pneumococcus in human pneumococcal challenge experiments. Among the proteins described above, another promising vaccine candidate is PspA. Previous studies have shown that it could be utilized as vaccine with both intranasal and subcutaneous administration, opening a broad range of developmental possibilities.

## Discussion

*S. pneumoniae* is an opportunistic commensal and is at the same time, one of the deadliest bacteria that has a long history of being in an inseparable relationship with humans. The development of antibiotics and vaccines is not yet sufficient to eliminate pneumococcal disease. To protect humans from pneumococcal disease, it is necessary to block pneumococcal transmission in addition to improving bactericidal measures or enhancing the immune mechanisms which confront pneumococcus after its entry into the body. Reducing its shedding, refining environmental hygiene, and blocking its acquisition, are all key.

In summary, this review focused on pneumococcal transmission because there has been great progress in research and understanding over the past decade due to the establishment of tractable animal models. The animal species used in these models are ferrets and mice. There are two major advantages of using ferrets in the models. First, they can transmit pathogens through droplets by sneezing like humans. Second, the glycomic profiles of ferrets are more similar to those of human than mice (Walther et al., 2013; Jia et al., 2014). Meanwhile, the accessibility to genetic engineering techniques such as transgenesis (e.g. humanized mice) and gene knock-out is one of the major advantages in murine models, and these techniques can be more utilized to clarify the specific genetic factors in pneumococcal transmission. There are several limitations of the current animal models of pneumococcal transmission. First, the influence of pneumococcal infection alone is difficult to evaluate in IAV coinfection models. Second, in the infant mouse model of pneumococcal monoinfection, shedding collection is a manual procedure and the result can be dependent on the technique of its measurer. Furthermore, the infant mouse model cannot mimic human adult pneumococcal transmission because the immune system of infant mice is immature. Moreover, the major route of transmission in murine models is thought to be contact transmission, while in humans is thought to be droplet transmission. Therefore, establishing a novel pneumococcal monoinfection model of adult mice with an objectively quantitative method of measuring the amount of shedding, is necessary for further research. Using these tractable animal models, it may be possible to evaluate the detailed effects of available vaccines on each transmission steps. Epigenetic analysis and the next generation sequencing such as RNA-seq and Tn-seq may reveal more dynamic mechanisms of pneumococcal infection and facilitate the search for vaccine candidates and therapeutic targets more readily. Further research on the comprehensive pneumococcal mode of life will enable us to improve our strategies for prevention and treatment of pneumococcal disease.

## Author Contributions

AM wrote the manuscript. SH and YA reviewed the manuscript. KT supervised this work. All authors contributed to the article and approved the submitted version.

## Funding

This work was supported by JSPS KAKENHI Grant Number 19K17924.

## Conflict of Interest

The authors declare that the research was conducted in the absence of any commercial or financial relationships that could be construed as a potential conflict of interest.
